# Editorial to the Special Issue “Vision Sensors: Image Processing Technologies and Applications”

**DOI:** 10.3390/s24061803

**Published:** 2024-03-11

**Authors:** Leandro A. F. Fernandes

**Affiliations:** Instituto de Computação, Universidade Federal Fluminense (UFF), Niterói 24210-346, Brazil; laffernandes@ic.uff.br

Computer vision and optical technologies have become attractive alternatives for metrology, chemical analysis, and non-destructive structural health monitoring and the evaluation of aerospace, civil, and mechanical engineering structures. The popularization of vision techniques for the creation of sensors for various purposes has mainly been driven by advancements in digital cameras, image processing algorithms, and deep learning approaches that have leveraged the development of new, reliable tools.

In order to unlock the complete potential of vision sensors, it is imperative to implement effective control and optimization strategies that leverage the ability to deal with the diversity of scenarios experienced on the wide. Collaboratively, the development of new computer vision technologies for sensors could also lead to the development of more appropriate sensors adapted to the new reality that is emerging with the advancement of computer vision techniques. This Special Issue aims to assemble original research and review articles on recent advancements, technologies, solutions, applications, and new challenges in vision sensors.

Manuscripts submitted to the Special Issue were subjected to at least two reviewing cycles with a rigorous peer-reviewing process. Six papers completed the reviewing process successfully. Their research topics include context-aware patch descriptors and context-aware patch matching [Contribution 1], tone-mapping and demosaicing [Contribution 2], the detection and recognition of tilted characters [Contribution 3], image segmentation [Contribution 4], the implementation of convolutional neural networks (CNNs) on image processor chips [Contribution 5], and emotion recognition [Contribution 6].

Sun et al. [Contribution 1] propose a modified patch-NetVLAD strategy with a new context-aware patch descriptor module and a context-aware patch-matching mechanism. They applied their approach to visual place recognition. Their work has the potential to pave the way for more effective solutions with applications in navigation, augmented reality, and robotics.

Automated driving systems (ADSs) can utilize high dynamic range (HDR) images to improve traffic safety. Following this trend, Stojkovic et al. [Contribution 2] developed two novel CNN architectures for the tone-mapping and demosaicing of HDR images with reconstruction quality focused on ADS object detection quality. The future impact of this work may be significant in improving the overall safety and reliability of automated driving systems by providing better visibility and recognition capabilities.

Xu et al. [Contribution 3] propose a deep learning-based approach that applies two recurrent neural networks to detect and recognize tilted characters on railroad wagon wheelsets. Xu et al.’s work has the potential to significantly impact railway safety, maintenance efficiency, operational optimization, technology transfer, and data-driven decision making.

Real-time segmentation in unstructured environments is necessary to enable autonomous navigation in off-road robots. To this end, Lin et al. [Contribution 4] propose a lightweight variant of the DDRNet23-slim model that includes a semantic-aware normalization and a semantic-aware whitening (SAN–SAW) module in the core network to improve the generalization ability beyond the visible domain. This work holds the possibility of leveraging the ability of existing robot navigation systems to handle diverse and challenging terrains by enhancing the ability of vision sensors to interpret complex and dynamic environments.

Lepecq et al. [Contribution 5] demonstrate how to implement an end-to-end CNN pipeline inside 3D-integrated focal-plane array image processor chips, achieving impressive frame rates on image classification by exploiting the different levels of parallelism available. The direct integration of CNN pipelines into image processor chips signifies a move towards edge computing in vision sensor technology. This can reduce reliance on centralized processing, enabling faster response times and increased autonomy for devices equipped with vision sensors.

A systematic review covering new trends in emotion recognition using neural network image analysis is presented by Cîrneanu et al. [Contribution 6] from historical and conceptual perspectives. This review can serve as a comprehensive resource for vision sensor researchers and developers, offering insights, benchmarks, and challenges that can inform the design and implementation of more advanced and effective emotion-aware vision sensors.

*Sensors* is committed to attracting high-quality works. The quality of the papers published in this Special Issue highlights this continuous effort.

